# “Incidental Papillary Thyroid Cancer in Thyroglossal Duct Cyst”: A Case Report

**DOI:** 10.22038/ijorl.2021.46922.2565

**Published:** 2021-05

**Authors:** José-Luis Saavedra-Leveau, Silvana-Lucia Chang-Grozo, Melissa-Elvita Dominguez-Prado, Luis-Alfredo Ticona-Zegarra

**Affiliations:** 1 *Head, Neck and Maxillofacial Surgery Service, Hospital Nacional Dos de Mayo, Lima-Perú, Universidad Nacional Mayor de San Marcos, Lima-Perú.*; 2 *Head, Neck and Maxillofacial Service, Hospital Central Policía Nacional del Perú.*; 3 *Head, Neck and Maxillofacial Service, Hospital Nacional de Salud del Niño, Breña.*

**Keywords:** Ectopic thyroid, Head and Neck cancer, Surgical management, Thyroglossal, Thyroid cancer

## Abstract

**Introduction:**

Papillary thyroid cancer (PTC) in thyroglossal duct cyst (TGDC) is an infrequent condition with less than two hundred cases described in literature, with an incidence likely to be close to 1%. While its management is quite straightforward, there exists significant controversy regarding whether additional treatment is needed to manage incidentally noticed carcinoma in TGDCs.

**Case Report:**

A 37-years-old man came to us complaining of a slowly progressive neck mass located in the midline from 4 years ago. Ultrasonography (US) showed a mixed tumor with cyst predominance of 90x79x50 mm and Computed-Tomography (CT) revealed a mixed inframentonian heterogeneous tumor associated with small, mostly peripheral calcifications. The mass was resected using Sistrunk’s surgery. Histologic review reported a moderately differentiated papillary carcinoma in thyroglossal duct cyst, without vascular and lymphatic invasion. After two months, a total thyroidectomy was done, to which the pathological report informed normal thyroid.

**Conclusion:**

Thyroglossal duct cyst carcinoma is a rare entity. Management should be decided on single risk stratification. In all cases, a Sistrunk surgery would be accomplished in order to remove the tumor. The reason for thyroidectomy in individuals with a normal thyroid is due to the probability of presenting an intraglandular thyroid cancer concomitantly. It also enables the management with radio-iodine and patient follow up by quantifying thyroglobulin levels.

## Introduction

Thyroglossal duct cysts (TGDC) are frequent inborn midline neck masses, nevertheless Papillary Thyroid Cancer (PTC) within these cyst is exceptionally uncommon, being less than two hundred cases described in literature (1). The real frequency of TGDC carcinoma (TGDCa) is almost near to 1% of all TGDC (2)**. **On the other hand, Ectopic Thyroid (ET) gland is described as thyroid mass located external to the 2nd to 4th tracheal cartilages (3).

The characteristics of TGDCa are similar to its benign equivalent regarding location, size or consistency^2^. Therefore, it is common that a diagnosis of carcinoma is not accomplished until after surgery^2^. However, malignancy must be considered in any tumor that is firm, fixed, irregular or accompanying with lymphadenopathies (2). 

The finding of a TGDCa often occurs as a surprise to both the patient and the treating physician (1). Although its treatment is quite simple, substantial debate exists referred to whether additional management is needed to treat incidentally discovered TGDCa (1). In this article we introduce a clinical case of this rare disease, as well as its respective management.

## Case Report

**Fig 1:**
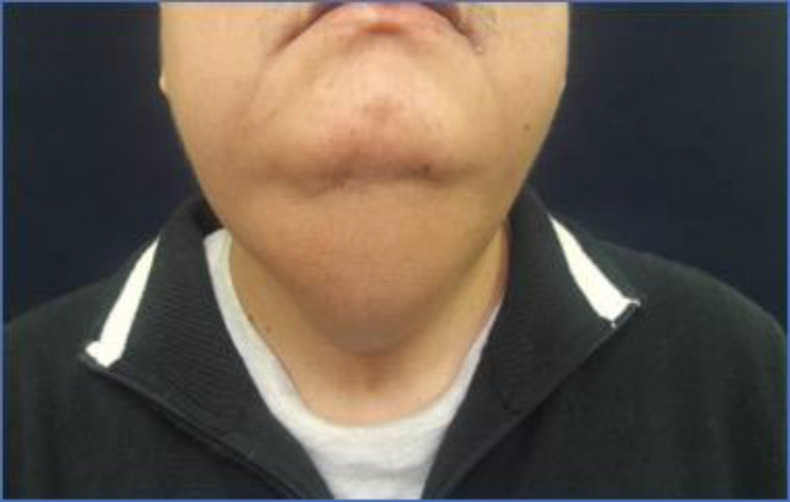
Preoperative photograph of the patient showing a midline tumor of the anterior cervical region of approximately 5x5 cm

Male of 37 years old who came to us complaining of a slowly progressive neck mass located in the midline which appeared 4 years ago associated with severe dysphagia. On physical examination, he presented a midline tumor of the upper and anterior cervical region of approximately 5x5 cm, of increased consistency, mobile, not adhered to deep planes, smooth surface and associated with mild pain at palpation (Figure.1).

**Fig 2:**
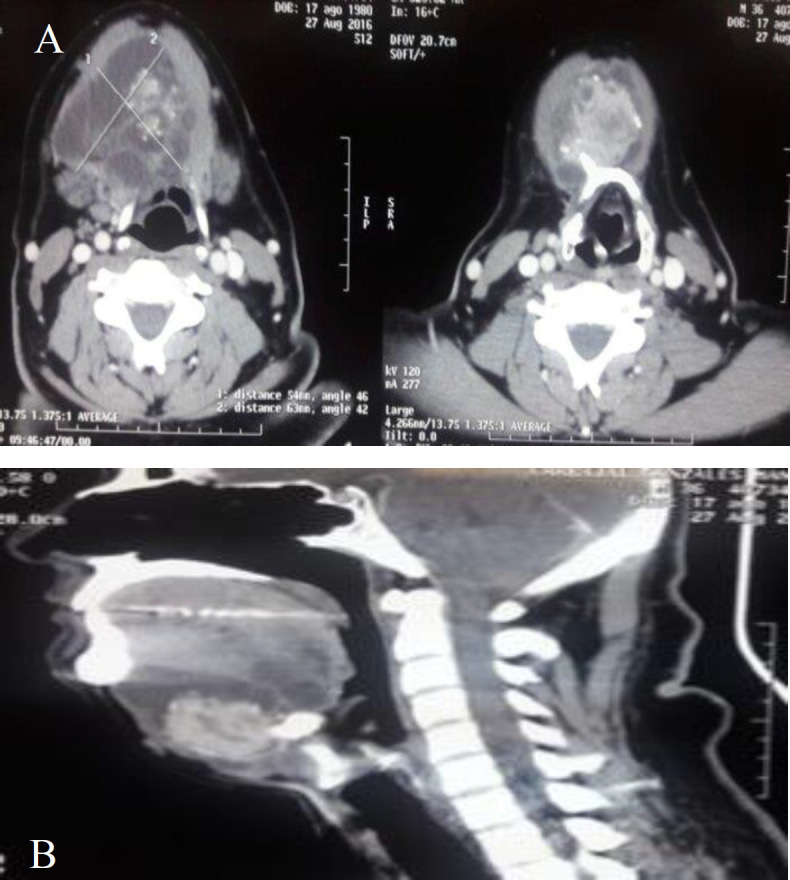
A. Contrast-enhanced computed tomography (CT) neck soft-tissue window axial view of patient that shows a mixed inframentonian heterogeneous tumor associated with small, mostly peripheral calcifications, that measured approximately 67x44x66 mm, conditioning compression of the aerodigestive tract B. Contrast-enhanced CT neck soft-tissue window sagital view of patient

Ultrasonography (US) showed a mixed tumor with cyst predominance of 90x79x50 mm, with a thyroid gland with colloid cysts of <1 cm while Computed-Tomography (CT) showed a mixed (solid and cystic) inframentonian heterogeneous tumor associated with small, mostly peripheral calcifications, that measured approximately 67x44x66 mm, conditioning compression of the aerodigestive tract with normal thyroid gland without nodes (Figure 2). Thyroid gland function was average and fine needle aspiration cytology (FNAC) informed lymphoid reaction. 

The performed surgical intervention was Sistrunk’s surgery (removal of the cyst plus hyoidectomy). Intraoperative findings were a multilobed soft tumor of 9x8x8 cm in the upper third of the midline neck, well defined, with platysma muscle adhesions and liquid content. In addition, an adenopathy of 1x1 cm in right IA group of increased consistency was found.

**Fig 3:**
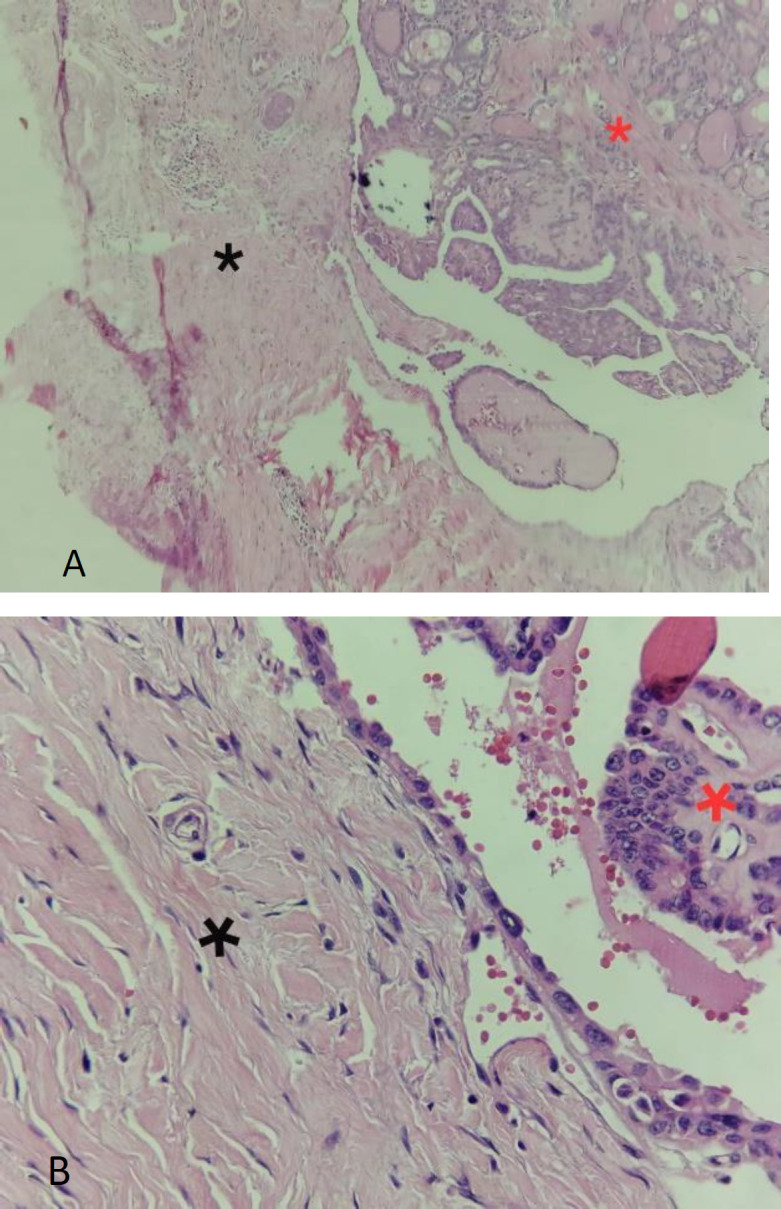
H&E stain at 20x (A) and 40x (B) magnification. Pathological figures show papillary cell carcinoma (red *) within thyroglossal duct cyst (black *)

The pathological report showed a moderately differentiated papillary carcinoma in TGDC, without vascular and lymphatic invasion (Figure.3). 

Two months later, a total thyroidectomy was completed and pathological report informed a normal thyroid. The patient had a favorable postoperative evolution, he is currently taking hormone replacement with levothyroxine and keeps returning to controls without presenting any new developments in his previous pathology. 

## Discussion

TGDC is an abnormality of development described in almost 7% of the people and presents as a residual thyroid tissue alongside its track of migration from the base of the tongue to the anterior neck (2). Of the people with a TGDC, only 1% develop a malignancy (4). TGDC is often present within pediatric population, and involving, in most of the cases, individuals under the age of 30; in contrast, TGDCa tends to be developed on the later stages of lifetime, occurring in an average age of 39.5 years (5). Most of malignancies in TGDC are papillary thyroid carcinomas; being the most unconventional ones follicular and squamous carcinomas (4). It has been proposed that they develop “*de novo” *from the duct remnants of the TGDC instead of secondary to thyroid gland malignant tumors (4). ET tissue can suffer from malignant transformation as well (6).

On ultrasonography, TGDC can appear as anechoic or hypoechoic. Malignancy manifests itself as a mural lesion inside the cyst, with microcalcification associated ocassionally (4). On CT and MRI, a solid nodule inside the cyst can be found (4). Some physicians endorse the regular practice of fine needle aspiration citology in all patients^4^. It is especially helpful if it samples positive for cancer (4).

Widstrom et al (7) described the diagnostic criteria of primary TGDCa: (1) the cancer must be present within the wall of the TGDC, (2) a differentiation from lymphatic metastasis by histological evidence must be realized and (3) no cancer in another primary site must be found. The ultimate criteria is still discussed, as it ignores approximately 11% to 40% of all TGDCa cases where it also exits a concomitant thyroid carcinoma (1). Currently, there is lack of standard recommendations about the ideal management of TGDCa. In addition, there is considerable controversy concerning the need of additional treatment for incidentally discovered TGDCa (1,2). However, it is recommended that a Sistrunk procedure should be realized to remove the lesion (5), regardless of the presence or suspicion of carcinoma (1).

Different opinions exist regarding whether patients with TGDCa must receive a thyroidectomy (2). Some specialists do not advise it in “low-risk” individuals with normal thyroid glands (4). The criteria for low-risk patients are: more than fifeteen and less than forty five years-old, no antecedent of radiation, less than four cm diameter without soft tissue extension or distant metastases (4).

Other specialists recommend a total thyroidectomy (4), mainly if another focus in the thyroid cannot be discarded. The main reasons for this indication in patients with a clinically normal thyroid is due to the increased probability (11–27%) of presenting a concomitant intraglandular thyroid cancer (4). It also enables radio-iodine treatment and allows the control for disease recurrence by quantifying thyroglobulin levels (4). Nevertheless, it should be considered that a thyroidectomy is related to increased morbidity (4), as hypocalcaemia and recurrent laryngeal nerve injury (4). Therapeutic lymphatic node dissection would be indicated in cases of suspicious of metastasis (4). However, its role is still controversial (4).

## Conclusion

TGDCa is a rare entity. Its presence in the background of a normal thyroid gland should be studied as a differential diagnosis in cases of an recognized neck mass. Early recognition and discrimination from other patholigies increase clinical outcomes. Management should be decided on single risk stratification. In all cases, a Sistrunk surgery would be accomplished in order to remove the tumor. The reason for thyroidectomy in individuals with a clinically normal thyroid is due to the possibility of presenting an intraglandular thyroid cancer concomitantly. It also enables the management with radio-iodine and allows the supervision for disease recurrence by quantifying thyroglobulin levels. This case report demonstrates that TGDCa can occur, so the physician must be prepared to diagnose and manage it.
